# Application of BiPAP through Endotracheal Tube in Comatose Patients with COPD Exacerbation

**DOI:** 10.12669/pjms.336.13972

**Published:** 2017

**Authors:** Nousheen Akhter, Nadeem Ahmed Rizvi

**Affiliations:** 1 Dr. Nousheen Akhter, FCPS trainee.Department of Chest Medicine, Jinnah Postgraduate Medical Centre Karachi, Pakistan; 2 Prof. Nadeem Ahmed Rizvi, FRCP.Department of Chest Medicine, Jinnah Postgraduate Medical Centre Karachi, Pakistan

**Keywords:** COPD, BiPAP, Respiratory failure

## Abstract

**Objective:**

To evaluate the effectiveness and safety of using BiPAP through endotracheal tube in comatose Chronic Obstructive Pulmonary Disease (COPD) patients with hypercapnic respiratory failure.

**Methods:**

This is a prospective study done at Department of Chest Medicine, Jinnah Postgraduate Medical Centre, Karachi, during March to June 2017. It included all comatose COPD patients with hypercapnic respiratory failure who had a poor functional status prior to the illness and who did not meet the criteria to be kept on mechanical ventilator. Patients with apnea and other causes of coma were excluded. These patients were applied BiPAP through endotracheal tube and its response on blood gases and neurological status was evaluated.

**Results:**

The success rate of BiPAP through endotracheal tube was 70.5% (31/44). Improvement in Glasgow Coma Scale (GCS) score (p<0.01), pH (p<0.01), and PaCO_2_ (<0.01) was observed among the responders following two hours and 24 hours of therapy. No significant difference was found in response with regards to gender, smoking status, prior use of noninvasive ventilation or duration of disease. No complications were observed during the therapy.

**Conclusion:**

In resource poor settings, the use of BiPAP through endotracheal tube can be an effective and safe intervention for comatose COPD patients with hypercapnic respiratory failure.

## INTRODUCTION

Chronic Obstructive Pulmonary Disease (COPD) is a leading cause of morbidity and mortality across the globe.^1^ Estimates from WHO’s Global Burden of Disease and Risk Factors project^2^ show that in 2001, COPD was the sixth leading cause of death in low and middle income countries, accounting for 4.9% of total deaths.

Respiratory failure is common in COPD exacerbation and the presence of hypercapnia is associated with significant mortality.^3^ Noninvasive positive pressure ventilation (NIPPV) is an effective way of managing respiratory failure associated with COPD.^4^ Prior studies suggest that NIPPV through BiPAP must be used as a first line treatment in these patients and it can effectively reduce the rate of endotracheal intubation and the complications related to invasive mechanical ventilation.^5^-^12^

Noninvasive positive pressure ventilation with face mask is contraindicated for unconscious patients, primarily because of the inability to handle secretions, that risks aspiration.^13^ Hence, intubation and mechanical ventilation remains the sole treatment modality for such patients. In Pakistan, the facility of mechanical ventilation and trained staff is not easily available especially in the remote areas. Where available, the cost is too high which is out of the reach of most patients.

Few studies^14^,^15^ done in past demonstrated that NIPPV with face mask can be used successfully in selected patients with varying degrees of consciousness. This study was performed to evaluate the effectiveness and safety of using BiPAP through endotracheal tube in comatose COPD patients with hypercapnic respiratory failure.

## METHODS

This is a prospective observational study which was performed at intensive care unit (ICU) of department of pulmonology, Jinnah Postgraduate Medical Centre, from March 2017 to June 2017. This is a 15-bedded ICU attended 24 hours by qualified pulmonologists and staffs. This study was approved by the hospital’s ethical committee. Written informed consent was obtained from the nearest accompanying relative of each patient.

Inclusion criteria of the study was all consecutive COPD patients with uncompensated hypercapnic respiratory failure (partial pressure of carbon dioxide [PaCO2] >45 mmHg and pH < 7.25), altered level of consciousness (Glasgow coma scale [GCS] score < 8), and who had contraindications to the use of NIPPV via facemask.

The exclusion criteria for the study were patients with apnea or those with altered level of consciousness due to causes other than respiratory failure including hypoglycemia, cerebrovascular accident and drug-induced coma. Patients for whom consent was not given for endotracheal intubation were also excluded.

Endotracheal tube was passed to all patients and was connected to NIPPV (BiPAP ST, ResMed) via connector tube. The BiPAP used had: (1) inspiratory positive airway pressure (IPAP), 2 – 40 cm H_2_O; (2) expiratory positive air way pressure (EPAP), 2 – 16 cm H_2_O; (3) breath rate, 5 – 60 breaths per minute; (4) timed inspiration, 0.1– 2.0 seconds; (5) rise time, 1%–6%; Patients were kept in a semi-recumbent position with the head raised at 45°. A nasogastric tube was inserted in all patients. Vital signs (non-invasive blood pressure monitoring, pulse, temperature, respiratory rate), electrocardiogram (ECG) and blood oxygen saturation (SpO_2_) were monitored continuously. GCS score was assessed two hours and 24 hours after BiPAP therapy. The BiPAP was set in the spontaneous/timed mode, with a backup respiratory rate of 15 breaths per minute. The initial IPAP and EPAP were set at 24 cmH_2_O and 12 cmH_2_O respectively with decrease in pressures if needed. Oxygen inhalation was adjusted to maintain SpO_2_ between 88% and 92%. Arterial blood gas (ABG) samples were obtained from each patient before starting and then 2 hours and 24 hours after BiPAP therapy.

Responders to the treatment were defined as patients who gained full consciousness after BiPAP therapy. Patients were labelled non-responders if at least one of the following occurred: (1) worsening of consciousness within two hours of initiating BiPAP; (2) deterioration of ABG, defined as no improvement or deterioration in pH, PaCO_2_, and partial pressure arterial oxygen (PaO_2_) from baseline measurement after two and 24 hours of BiPAP administration; (3) respiratory or cardiac arrest or (4) development of hemodynamic instability.

Midazolam was used if required in agitated patients. Patients were extubated once they gained full consciousness and thereafter were given BiPAP through facemask.

Statistical analysis was performed using SPSS Statistics (IBM Corporation) software, version 23.0. Mean and standard deviation were calculated for quantitative variables. Chi square test was used to evaluate the significance of difference between categorical data. Frequencies were calculated for quantitative variables. Comparison of the monitored variables between responders and non-responders was done using repeated measures ANCOVA (analysis of covariance), keeping confidence interval 95% and p-value <0.05.

## RESULTS

A total of 44 patients met the inclusion criteria of the study. Out of these 31 (70.5%) patients responded to the therapy while 13 (29.5%) patients were non-responders. There was no significant difference between the baseline characteristics including age, gender, smoking status, duration of COPD, prior use of non-invasive ventilation, vital signs and conscious level of both the groups: responders and non-responders, as shown in Table-I.

**Table-I:** Baseline characteristics of study population.

*Variables*	*Responders (n=31)*	*Non-responders (n=13)*	*p-value*
Gender (male/female)	26/5	10/3	0.58
Age (years)	63.48 ± 11.61	65.77 ± 9.50	0.53
Duration of COPD (years)	4.77 ± 3.15	6.31 ± 3.40	0.15
***Smoking status:***			
Non-smoker	6	2	0.92
Smoker	8	4	
Ex-smoker	17	7	
Prior use of NIV (yes/no)	10/21	8/5	0.07
pH	7.10 ± 0.10	7.11 ± 0.18	0.96
PaCO_2_ (mmHg)	106.41 ± 26.54	88.23 ± 39.51	0.08
PaO_2_ (mmHg)	75.14 ± 36.24	72.49 ± 27.15	0.81
Systolic BP (mmHg)	120 (70 – 170)	110 (60 – 190)	0.49
Heart rate (beats per minute)	108 (44 – 140)	96 (60 – 130)	0.09
Respiratory rate (breaths per minute)	32 (12 – 40)	26 (8 – 48)	0.78
GCS	4 (3 – 13)	5 (3 – 13)	0.99

Mean and standard deviation are shown for age, duration of COPD, pH, pCO_2_ and pO_2_. Median and range are shown for systolic BP, heart rate, respiratory rate and GCS. PaCO_2_: partial pressure of carbon dioxide, PaO_2_: partial pressure of oxygen, BP: blood pressure, GCS: Glasgow Coma Scale

While analyzing the pair wise progressive course of the monitored parameters, a statistically significant improvement was observed in pH (p-value <0.01), PaCO_2_ (p-value <0.01) and GCS (p-value <0.01) among the responders as compared to the non-responders, which was maintained throughout the study period. The results of ANCOVA indicating a significant effect of therapy with BiPAP applied through endotracheal tube, when calculated for pH, PaCO_2_ and GCS is shown in Table-II.

**Table-II:** Comparison of ABG variables and GCS between responders and non-responders with BiPAP therapy.

*Variables*	*Baseline*	*At 2 hours*	*At 24 hours*	*p-value*
***pH:***				
Responders	7.10 ± 0.10	7.27 ± 0.09	7.36 ± 0.05	<0.01
Non-responders	7.11 ± 0.18	7.18 ± 0.11	7.22 ± 0.11	
***PaCO_2_ (mmHg):***				
Responders	106.41 ± 26.54	74.43 ± 16.22	56.26 ± 10.80	<0.01
Non-responders	88.23 ± 39.51	86.31 ± 42.81	73.50 ± 34.66	
***PaO_2_ (mmHg):***				
Responders	75.14 ± 36.24	74.01 ± 15.30	70.49 ± 12.91	0.84
Non-responders	72.49 ± 27.15	71.98 ± 13.42	72.28 ± 14.68	
***GCS:***				
Responders	5.84 ± 3.16	8.84 ± 1.69	14.87 ± 0.42	<0.01
Non-responders	5.85 ± 2.82	5.77 ± 1.83	5.00 ± 2.00	

PaCO_2_: partial pressure of carbon dioxide, PaO_2_: partial pressure of oxygen, GCS: Glasgow Coma Scale.

The PaO_2_ for both the groups was maintained at an acceptable level from baseline at two hours and 24 hours but the difference was not statistically significant (p-value 0.84).

## DISCUSSION

To the best of our knowledge, apart from a small pilot study^16^ done in India, this is the only study reporting the use of BiPAP therapy through endotracheal tube in unconscious COPD patients. This therapy was seen successful in improving gas exchange and neurological status of majority (70.5%) of the patients.

Decreased consciousness is known as a contraindication to the use of NIPPV in general because it is thought that this therapy does not help the uncooperative patient and it is also not safe because of the risk of pulmonary aspiration and difficulty in managing bronchial secretions mainly because of depressed cough reflex.^17^,^18^ There is a very limited data on the use of non-invasive positive pressure ventilation by face mask in unconscious patients. A study done by Scala et al^14^ demonstrated better outcome of NIPPV in patients with low level of consciousness. Zhu et al^19^ also showed in their small study 84.61% success rate of NIPPV in comatose COPD patients. Another small study conducted in 1992 by Benhamou et al^20^ also showed that NIPPV can be safely used in unconscious patients (60% success rate). Dueñas-Pareja et al^21^ reported a hospital survival rate of 69% after treatment with NPPV using a facial mask in 13 ARF patients (mean pH, 7.17) who were in hypercapnic coma (GCS, ≤ 7) and were not candidates for ICU admission. A few case reports^22^,^23^ have also shown successful use of NIPPV with face mask in comatose state.

In our study, we used a different method of administering BiPAP. Instead of using face mask, we used endotracheal tube as an interface between patient and the machine. In general, inserting an endotracheal tube to an unconscious patient reduces the risk of aspiration and blockade of the upper airway by tongue falling behind. Approximate 2/3^rd^ success rate of this therapy as shown in our study is likely due to reduction of the risk of aspiration and pneumonia. By using face mask there is a risk of leakage of inspiratory pressures through the potential air spaces between skin and the ill-fitting face mask. The chances of this leakage are minimized by endotracheal tube.

A significant change was observed in pH, PaCO_2_ and GCS of the patients who responded to the therapy. Follow up comparisons indicated that each pairwise difference was also significant, p-value <0.01. There was a significant improvement in the aforementioned parameters with time, such that responders can be identified earlier during therapy. Non-responders showed progressive deterioration in all parameters. Although this is a small study, the promising results extracted from this study merit further validation in a randomized controlled trial.

## CONCLUSION

In resource poor settings, the use of BiPAP through endotracheal tube can be an effective and safe intervention for comatose COPD patients with hypercapnic respiratory failure.

### Authors’ Contribution

**NAR** conceived, designed and reviewed the manuscript.

**NA** did data collection, statistical analysis and manuscript writing.
